# Sonographic characteristics of thyroid nodules with a Halo

**DOI:** 10.1186/s13044-024-00208-5

**Published:** 2024-10-01

**Authors:** Danming Cao, Rong Zou, Ming Zhang, Kui Tang

**Affiliations:** 1 Department of Ultrasound Diagnosis, The Second Xiangya Hospital, Central South University, Changsha, Hunan 410011 China; 2 Research Center of Ultrasonography, The Second Xiangya Hospital, Central South University, Changsha, Hunan 410011 China; 3 Clinical Research Center for Ultrasound Diagnosis and Treatment in Hunan Province, Central South University, Changsha, Hunan 410011 China; 4 Department of Gynecology and Obstetrics, The Second Xiangya Hospital, Central South University, Changsha, Hunan 410011 China; 5 Department of Ultrasound and Electrocardiography, Guilin Hospital of the Second Xiangya Hospital, Central South University, Guilin, Guangxi 541006 China

**Keywords:** Thyroid nodules, CEUS, FNA, Halo, Metastasize

## Abstract

**Objective:**

To investigate the sonographic characteristics of thyroid nodules with a halo, explore the value of contrast-enhanced ultrasound (CEUS) combined with fine needle aspiration (FNA) in identifying nodules with a halo, and predict the risk of metastasis by analyzing the pathological features of the halo.

**Methods:**

A retrospective analysis was conducted on 185 postoperative cases of thyroid nodules accompanied by halos between January 2019 and December 2022. After describing the ultrasound characteristics of the thyroid nodules and their halos, all patients were divided into three groups, the first group (group I = CEUS only) of patients underwent CEUS, the second group (group II = CEUS + FNA) underwent FNA based on the first group, and the third group (group III = FNA only) underwent FNA directly. The CEUS and FNA results were graded using the Chinese Thyroid Imaging Report and Data System (C-TIRADS) and Bethesda Reporting System for Thyroid Cytopathology, respectively. Those graded below C-TIRADS 4b or Bethesda IV were defined as benign, and the results of FNA were referenced when the two methods were combined. The surgical pathology results were used as the gold standard. We plotted working curves to compare the diagnostic efficacy of CEUS and FNA alone and in combination in the diagnosis of thyroid nodules with halos. The pathological features of the halo were analyzed and the number of patients with cervical lymph node metastases was recorded.

**Results:**

One hundred and sixty patients met the requirements. Benign nodules were mainly characterized by a thin (0.75 ± 0.31 mm) and uniform halo with good integrity, while malignant nodules had a thicker (1.48 ± 0.51 mm) halo with uneven and irregular margins (*P* < 0.05). The sensitivity and specificity were highest when the cutoff value was 1.09 mm, with 76.08% and 84.29%, respectively. The halos of benign nodules were mostly hyper- or iso-enhanced, whereas the halos of malignant nodules were predominantly hypo-enhanced (*P* < 0.05). The areas under the curve (AUCs) for CEUS, FNA, and CEUS + FNA were 0.751(95% CI = 0.642–0.841), 0.863(95% CI = 0.767–0.929), and 0.918(95% CI = 0.834–0.967), respectively. Cervical lymph node metastasis occurred in only 13 (11.5%) malignant nodes with halos. The primary pathological components of the halo around malignant nodules were almost reactive hyperplastic fibrous tissue.

**Conclusion:**

The halo surrounding malignant thyroid nodules is thicker, with uneven and irregular margins, and shows hypo-enhancement on CEUS. Combining CEUS with FNA improves the diagnostic efficacy of thyroid nodules with halos. The reactive hyperplastic fibrous halo may be one of the reasons why malignant nodules are less likely to metastasize.

**Supplementary Information:**

The online version contains supplementary material available at 10.1186/s13044-024-00208-5.

## Introduction

The incidence of thyroid cancer is rising globally. According to the GLOBOCAN 2020 database of cancer incidence and mortality published by the International Agency for Research on Cancer of the World Health Organization, thyroid cancer ranks ninth in terms of cancer incidence worldwide [1, 2]. Moreover, the incidence rate is trending toward younger populations. The early and accurate diagnosis of thyroid cancer holds significant importance.

Ultrasound is the most commonly used method for diagnosing and differentiating thyroid nodules [3–7]. The latest Thyroid Imaging Report and Data System (TIRADS) diagnostic guidelines list solid composition, markedly hypoechoic, irregular margin, microcalcification, taller than wide, and other internal structure evaluations as the main indicators for diagnosing malignant thyroid nodules [8]. However, there are few studies on the ultrasound characteristics of structures surrounding thyroid nodules, such as the halo, and reports on the pathological features of the halo and predicting the possibility of metastasis to cervical lymph nodes based on the halo are even rarer. This study retrospectively analyzed the traditional and CEUS characteristics of 160 thyroid nodules and their halos, and summarized the pathological features of halos. The aim is to improve the diagnosis of benign and malignant thyroid nodules and predict the risk of cervical lymph node metastasis in malignant nodules based on the characteristics of the halo.

## Materials and methods

### Participants

The study was conducted according to the Declaration of Helsinki and approved by the Ethics Committee of the Second Xiangya Hospital of Central South University. All patients signed an informed consent form before CEUS examination and needle biopsy. A total of 185 patients with solitary nodules accompanied by acoustic halos were enrolled from January 2019 to December 2022. The inclusion criteria were as follows:

1. Isolated thyroid nodules;
2. Nodules with a halo;
3. Thyroid nodules with surgical pathological diagnosis.

The exclusion criteria were as follows:

1. multiple nodules;
2. patients allergic to contrast agents;
3. postoperative recurrence of thyroid cancer;
4. cysts of the thyroid;
5. parathyroid or thyroid glossal duct;
6. unilobular thyroid hypoplasia leading to contralateral lobe hyperplasia;
7. benign thyroid nodules formed by scars and hyperplasia of residual thyroid tissue after surgery or I^131^ treatment.

**Fig. 1:**
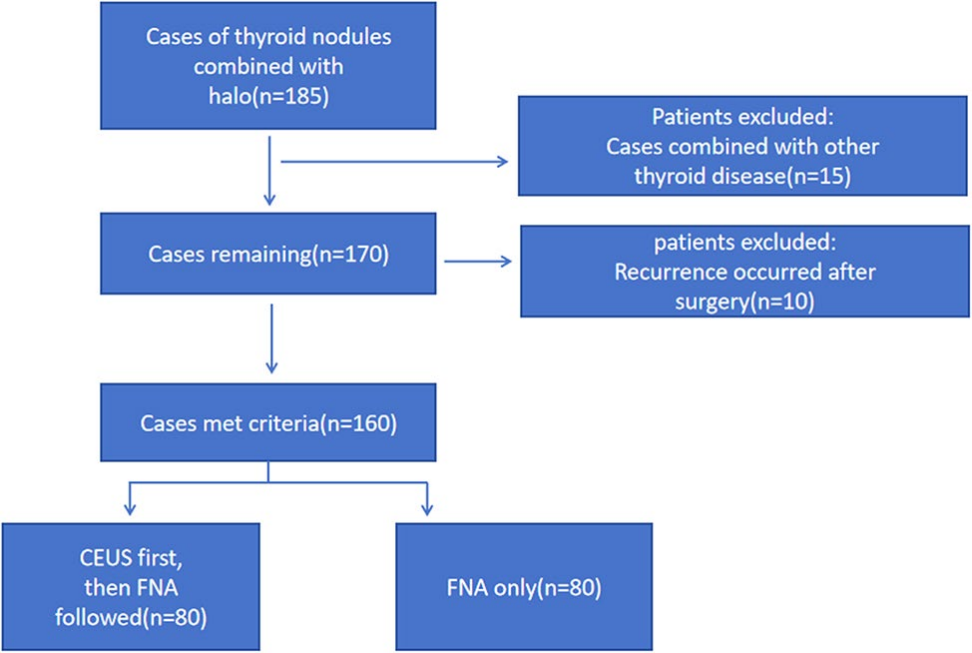
Flowchart of the present study

Ultimately, 160 patients were enrolled in the study (Fig. 1). The patients were divided into three groups and data were collected on the characteristics of traditional ultrasound and CEUS of thyroid nodules and their halos. The data included information on the location, size, margins, shape, internal echogenicity, calcification, blood flow distribution, and enhancement patterns of the thyroid nodules, as well as the thickness, regularity, integrity, blood flow distribution, and enhancement of the halo.

### Ultrasound imaging

#### Routine ultrasound examination

A Siemens ACUSON S3000 US scanner with a 9L4 (4–9 MHz) linear array transducer was used for routine US and CEUS examinations. The ultrasound image data were saved onto the instrument’s hard disk. During the examination, the patient was instructed to lie in the supine position with a pillow behind the neck so that the neck as in a hyperextended position to fully expose the anterior cervical region. When scanning one side of the thyroid gland, the patient could have been asked to turn his or her head to the opposite side if necessary. The thyroid glands were examined bilaterally and in the isthmus in both transverse and longitudinal views, taking care not to miss the conical lobes. In the conventional ultrasound mode, each nodule and the halo around it is observed by the same senior physician with more than 5 years of experience, with particular attention to halo characteristics such as thickness, regularity, clarity, and blood flow, and the findings were recorded. Measure at the thickest point when the halo is not regularity. According to the C-TIRADS guidelines there are five indications of malignancy: specifically solid composition, markedly hypoechoic, irregular margin, microcalcification, taller than wide, of these, 1 point for each indicator, and score 1 for category 4a, 2 for category 4b, 3 or 4 for category 4c, and 5 for category 5. Additionally, the bilateral cervical lymph nodes were routinely scanned for morphology, long/short ratio, calcification, hyperechoic mass, and liquefaction.

#### CEUS assessment

For the CEUS group, the CEUS mode was switched after routine ultrasonography using the same parameters (MI: 0.08) for all patients, and the focus was adjusted to the posterior aspect of the nodule. The probe was kept stable to ensure clear visualization of the nodule, and patients were instructed to avoid swallowing or talking. A total of 2.4 mL of SonoVue (Bracco, Italy) contrast agents was injected through the elbow vein, followed by a rapid saline flush of 5 mL. A timer was started immediately after injection of the contrast agent, and dynamic images were recorded for approximately 2 min and then saved to the hard disk and imaging workstation. Each nodule was graded according to C-TIRADS, taking into account the conventional ultrasound characteristics of the nodule. After completing the CEUS examination, FNA was performed in all patients.

#### FNA and surgery

Before the FNA procedure, a detailed medical history was taken, the risks and precautions were explained, and an informed consent form was signed. It was also explained to the patient that too small a sample might not be sufficient to support a cytological diagnosis or might lead to false-negative and false-positive rates. Patients of group II and III underwent FNA under ultrasound guidance, with a 22-gauge needle used for each nodule. Conventional smears were produced for each pass, stained with Diff Quik, and immediately analyzed under the microscope. The slides were fixed in 95% alcohol for pathological examination, with additional smears fixed in 95% ethanol for further Papanicolaou staining. Evaluation of the smears was based on the latest version of the Bethesda System [7]. According to the 2023 Bethesda System for Reporting.

Thyroid Cytopathology, FNA Bethesda cytology (BC) diagnoses were divided into six categories as follows: (i) nondiagnostic; (ii) benign; (iii) atypia of undetermined significance (AUS); (iv) follicular neoplasm; (v) suspicious for malignancy (SFM); and (vi) malignant. All patients underwent thyroid surgery, and the surgical specimens of all nodules were labeled, with the location of the halo around the nodule specifically marked and sent for pathological sampling, following the criteria of the latest version of the WHO classification [8]. Lymph nodes in the central cervical VI region were routinely cleared, and if the lateral cervical lymph nodes were diagnosed as metastatic, lateral cervical lymph node dissection was performed. The histopathological results were used as the only reference standard for the final diagnosis of malignant thyroid nodules and metastatic lymph nodes.

### Statistical analysis

Statistical analysis was performed by using SPSS 25.0. Collected data are presented as the mean ± SD for continuous variables with a normal distribution. The comparison between the benign and malignant groups was performed by the χ^2^ test. Using histopathology as the “gold standard”, the sensitivity, specificity, accuracy, positive predictive value (PPV) and negative predictive value (NPV) of CEUS and FNA in the diagnosis of benign and malignant thyroid nodules were calculated. The difference was statistically significant with *P* < 0.05.

## Result

A total of 160 eligible patients were included in this study. The clinical characteristics of the patients are shown in Table 1. There were 44 males and 116 females ranging in age from 19 to 66 years with a mean age of 42.1 ± 10.4 years. The mean age was 42.1 ± 10.4 years in the malignant group and 43.5 ± 10.5 years in the benign group. A total of 110 cases were surgically confirmed to be malignant, and 50 were benign. Of the patients with malignant lesions, 86 (78.2%) were female and 24 (21.8%) were male, while in the benign group, 30(60%) were female and 20 (40%) were male. Seventy-five cases (68.2%) of malignant nodules were hypoechoic and 42 cases (84%) of benign nodules were iso- or hyperechoic, with a statistically significant difference between the two groups (*p* < 0.05). Malignant nodules were predominantly ill-defined (89.1%), taller than wide (83.6%), microcalcified (56.4%), distributed with punctate blood flow (86.4%), had hypo-enhancement (80.8%) and had heterogeneous enhancement (88.5%), which were significantly higher than those in the benign group (*P* < 0.05).

**Table 1:** Comparison of the results of CEUS, FNA alone and the combination of the two methods in the diagnosis of thyroid benign and malignant nodules

Methods	Results		Pathology		
			Malignant	Benign	Total
CEUS	Malignant		41	11	52
	Benign		8	20	28
	Total		49	31	80
FNA	Malignant		50	8	58
	Benign		2	20	22
	Total		52	28	80
CEUS + FNA	Malignant		49	3	52
	Benign		3	25	28
	Total		52	28	80

**Fig. 2:**
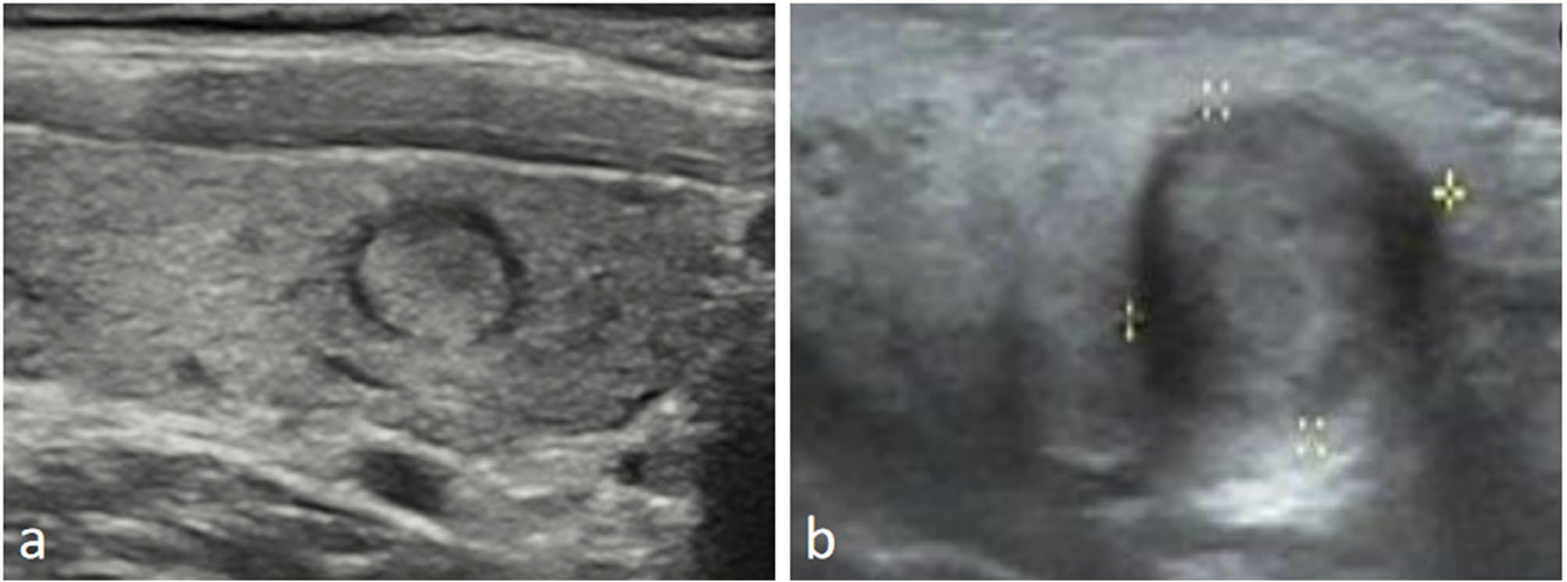
(**a**), A thin and complete hypoechoic halo is visible around the benign nodule. (**b**), The boundary of the halo around the malignant nodule is blurred, and the halo is thick and uneven in thickness

**Fig. 3:**
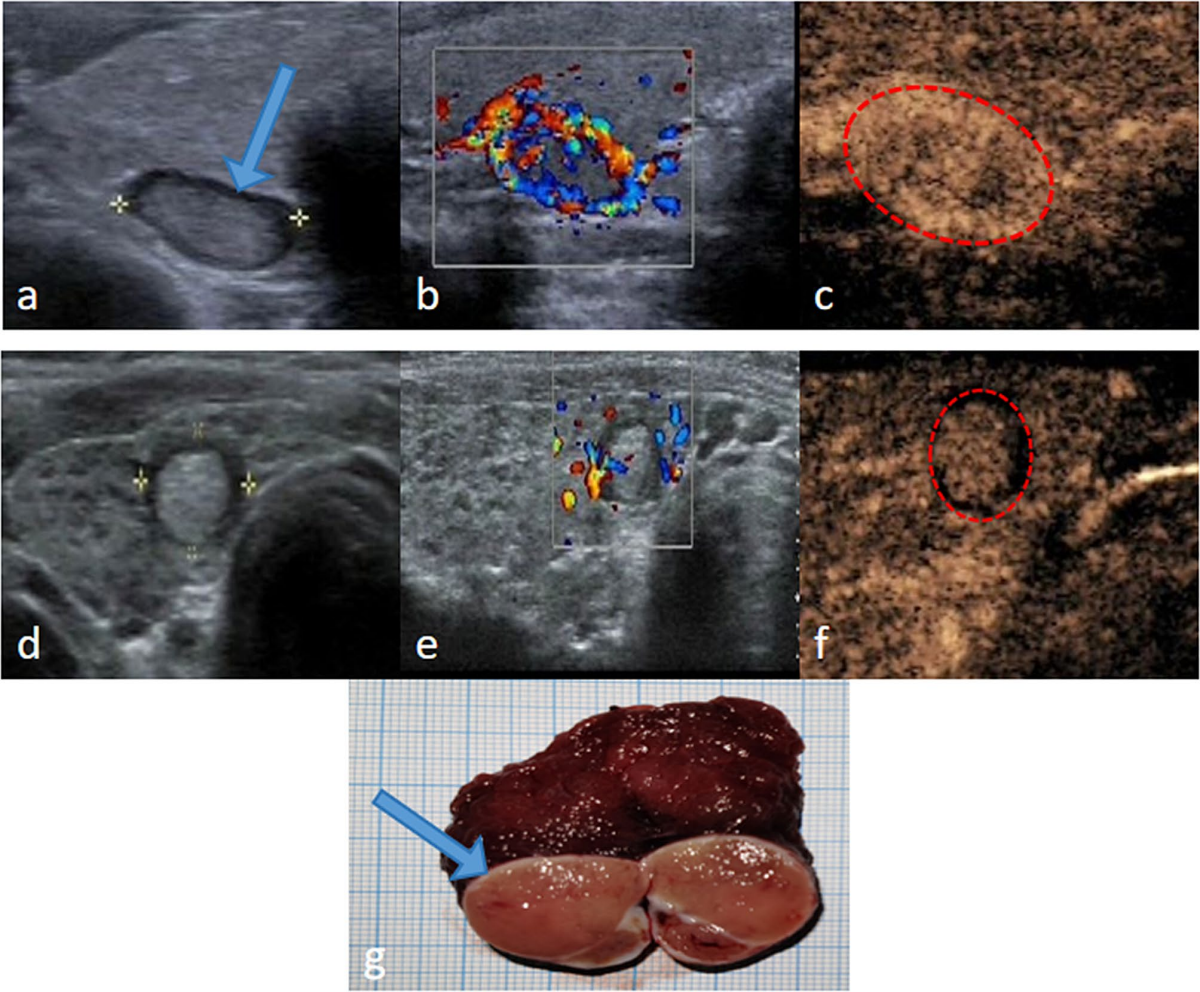
(**a**), A thin and complete hypoechoic halo around the nodule. (**b**), Color Doppler shows ring-like blood flow at the halo. (**c**), CEUS shows a distinct ring hyperenhancement of the halo (dashed line). (**d**), The boundary of the halo around the nodule is blurred, thick and uneven. (**e**), Only dotted blood flow signal is visible at the halo. (**f**), The halo showed hypo-enhancement on CEUS (dashed line). (**g**) Surgical specimen of the nodule, both the arrow indicates the position of the halo. **a-c**: Ultrasound presentation of the same benign nodule, **d-f**: Ultrasound of the same malignant nodule. **g**: Surgical specimen from Fig. 3a

There were 103 (64.4%) cases of 160 nodules with halo thickness greater than 1 mm, of which 92 (83.6%) were malignant. The mean thickness of the halos of the malignant and benign lesions were 1.48 ± 0.51 mm (range: 0.29–2.64 mm) and 0.75 ± 0.31 mm (range: 0.31–2.21 mm), respectively (*P* < 0.001). The halos of the benign nodules were even (64.3%) with good integrity (78.6%) (Fig. 2a). In contrast, the malignant nodules exhibited an uneven halo (84.6%) with poor integrity (90.4%) (Fig. 2b), and the difference between the two was statistically significant (*P* < 0.05). Among the malignant nodules, most of the halos had only a few dotted blood flow signals (92.7%) and showed hypo-enhancement (98.1%) on CEUS, while in the benign nodules, up to 20% of halos had circular blood flow signals and showed hyper- or iso-enhancement (67.9%) on CEUS, and the difference from malignant nodules was statistically significant (*P* < 0.05) (Fig. 3).

**Fig. 4:**
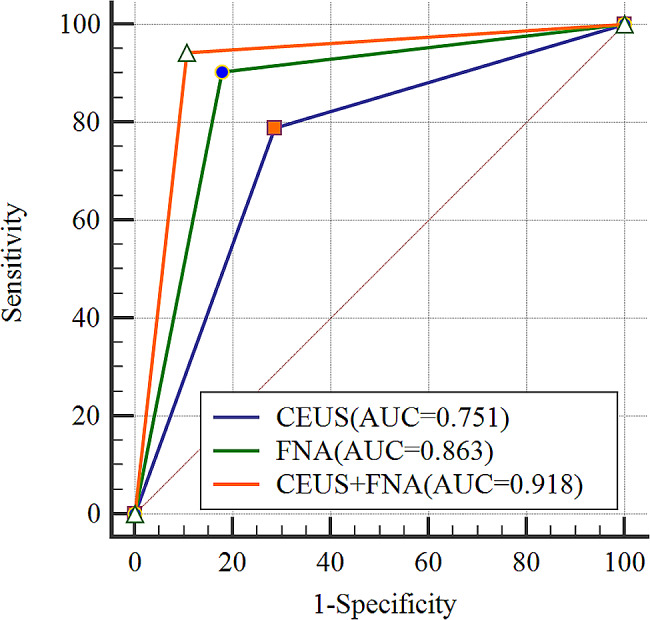
Diagnostic efficacy of CEUS, FNA and CEUS + FNA

The diagnostic results for TIRADS and Bethesda in both groups are shown in the Supplementary Tables 1 and 2. Of the approaches, CEUS correctly diagnosed 20 benign and 41 malignant cases (Table 2), and the sensitivity, specificity, accuracy, positive predictive value and negative predictive value of CEUS in diagnosing malignant thyroid lesions were 83.7%, 64.5%, 76.3%, 78.9% and 71.4%, respectively (Table 3). FNA correctly diagnosed 20 benign and 50 malignant cases (Table 2), and the sensitivity, specificity, accuracy, positive predictive value and negative predictive value of FNA in diagnosing malignant thyroid lesions were 86.2%, 90.9%, 87.5%, 96.2% and 71.4%, respectively (Table 3). Surprisingly, CEUS correctly combined with FNA diagnosed 25 benign and 49 malignant cases (Table 2), and the sensitivity, specificity, accuracy, positive predictive value and negative predictive value of CEUS in diagnosing malignant thyroid lesions were 94.2%, 89.3%, 92.5%, 94.2% and 89.3%, respectively, which were higher than those of CEUS and FNA (*P* < 0.05) (Table 3). The diagnostic efficacy of CEUS, FNA, and CEUS + FNA is shown in Fig. 4, with AUCs of 0.751 (95% CI = 0.642–0.841), 0.863 (95% CI = 0.767–0.929), and 0.918 (95% CI = 0.834–0.967), respectively.

**Table 2:** Comparison of diagnostic value of CEUS, FNA and combination of two methods

Methods	Sensitivity	Specificity	Accuracy	PPV	NPV	*P* Value
CEUS	87.3	64.5	76.3	78.9	71.4	< 0.05^a^
FNA	86.2	90.9	87.5	96.2	71.4	< 0.001^b^
CEUS + FNA	94.2	89.3	92.5	94.2	89.3	< 0.001^c^

PPV = positive predictive value; NPV = negative predictive value;

^a^: CEUS vs. FNA

^b^: FNA vs. CEUS + FNA

^c^: CEUS vs. CEUS + FNA

**Table 3:** Surgical pathology results in each group

	Pathological Typing	Group I	Group II
Benign			
	Nodular goiter	19	15
	Thyroid adenoma	3	1
	Benign follicular hyperplasia	4	3
	Fibrosis with chronic inflammatory cell infiltration	2	3
Malignant			
	Papillary thyroid carcinoma	49	55
	Thyroid follicular carcinoma	1	0
	Medullary thyroid carcinoma	2	3
Total		80	80

The surgical pathological findings of the two groups are shown in Table 4. In both groups, benign nodules were mainly nodular goiter and malignant nodules were mainly papillary thyroid carcinoma. The pathological features of the halo around the benign nodules are mainly compressed thyroid tissue (24%), fibrous connective tissue (52%), and vessels (16%). In contrast, the main pathological components of the halo around malignant tumors are infiltrating inflammatory cells (10%) and responsive proliferating fibrous tissue (81%) (Table 5). Of the 110 malignant nodules, only 13 cases of metastasis occurred.

**Table 4:** Pathological components of halo

	Compressed thyroid tissue	Fibrous connective tissue	Vessels	Infiltrating inflammatory cells	Reactive hyperplastic fibrous tissue	Total
Benign	12 (24%)	26 (52%)	8 (16%)	2 (4%)	2 (4%)	50
Malignant	4 (3.6%)	4 (3.6%)	2 (1.8%)	11 (10%)	89 (81%)	110

## Discussion

The present study investigated the sonographic characteristics of thyroid nodules with halos. The findings show that benign nodules were mainly characterized by a thin and uniform halo with good integrity, while malignant nodules had a thicker halo with uneven and irregular margins. The halos of benign nodules were mostly hyper- or iso-enhanced, whereas the halos of malignant nodules were predominantly hypo-enhanced. The AUC for CEUS, FNA, and CEUS + FNA were 0.751 (95% CI = 0.642–0.841), 0.863 (95% CI = 0.767–0.929), and 0.918 (95% CI = 0.834–0.967), respectively. The primary pathological components of the halo around malignant nodules were reactive hyperplastic fibrous tissue, and cervical lymph node metastasis occurred in only 13 malignant nodes with halos.

Thyroid cancer is receiving increasing attention as the incidence of thyroid cancer is gradually increasing. Ultrasound is used as an important tool for routine screening of thyroid disease. With the standardization of ultrasonography, an expert consensus has been formed on the ultrasonographic manifestations of thyroid cancer. However, in recent years, the acoustic halo of thyroid nodules has attracted much attention [9].

Halo refers to the hypoechoic zone around thyroid nodules. To date, there is still disagreement regarding the halo around thyroid nodules in helping to diagnose the nature of thyroid nodules [10–14]. Propper et al. reported that halos can occur around benign and malignant nodules, and may be a nonspecific sign of thyroid nodules [15]. The main reason for its formation is still unclear. A reasonable explanation is that, owing to the pathological tissue transition layer between the tumor and normal thyroid tissue, with the continuous growth of the tumor, the transition layer is continuously compressed, and the acoustic impedance value around the tumor is gradually transitioned. When the adjacent acoustic impedance difference in the transition layer is very small, the reflected and scattered signals of the ultrasonic wave are very small, and the phenomenon of a hypoechoic halo appears [16]. Clark et al. believed that the acoustic halo is caused by the vessels surrounding the adenoma [17, 18].

At present, few studies have elaborated on the traditional ultrasound and CEUS features of the halo, and studies summarizing the pathological features of the halo and predicting the risk of metastasis of malignant thyroid nodules are even rarer. Zhang [19] et al. found that the halo of malignant nodules is thicker and more irregular than that of benign nodules. In this study, we found that benign and malignant nodules have different halo appearances. The periphery of benign nodules is mainly composed of thin and uniform halos, with good integrity. The mean thickness of the benign nodule halo was approximately 0.75 ± 0.31 mm, which is consistent with the research results of Zhang [19]. The sensitivity and specificity were highest when the cutoff value was 1.09 mm, with 76.08% and 84.29%, respectively. This is because most thyroid adenomas have a complete fibrous capsule, resulting in blood vessels circulating under the capsule to form a thin and uniform halo. However, it was also found in this study that among the 50 benign nodules with halos, there were still 8 cases with irregular halos, accounting for approximately 16%. The reason for these conditions is that nodular goiter and follicular hyperplasia are proliferative disorders, and because the nodules are in different stages, hyperplasia squeezes the surrounding thyroid tissue, leading to pathological factors such as heterogeneity and atrophy of the halo. There is often extruded thyroid follicular tissue within the halo, and nodular goiter changes outside the halo rather than just compression of the atrophied thyroid gland, so the separation from the surrounding thyroid tissue is not clear [20], which manifests as an incomplete or irregular halo. This may also be one of the reasons why some nodular goiters are difficult to distinguish from thyroid cancer. Excitingly, in this study, it was found that the halos of malignant thyroid nodules were thicker and unevenly distributed in thickness, with a mean thickness of approximately 1.48 ± 0.51 mm, which was significantly thicker than that of benign nodules at 0.75 ± 0.31 mm (*P* < 0.05). The sensitivity and specificity of the diagnosis of malignant nodules were highest when the cutoff value was taken as 1.09 mm, with 85.45% and 80%, respectively. This is due to the aggressive growth of malignant tissues and the uneven distribution of neovascularization during tumor growth causing the nodules to grow in different directions. In the area with abundant neovascularization, the tissue grows faster and exerts more pressure on the surrounding tissues, and the inflammatory response is heavier, resulting in a thicker halo. In contrast, in areas with less neovascularization, the tumor grows relatively slowly, presses less on the surrounding tissue, and has a mild inflammatory response, so the halo is thinner and even disappears.

CEUS is a pure blood pool imaging technology that can dynamically observe the hemodynamic changes in thyroid nodules and their halos in real time. Although the value of CEUS is not confirmed in the TIRADS guidelines, it is generally accepted that CEUS has a positive role in the diagnosis of thyroid nodules [21–24]. In the present study, we further investigated the CEUS characteristics of halos. The halo of both benign and malignant nodules was predominantly hypo-enhanced, but 20% of benign nodules still showed ring enhancement. Based on this, 20 benign cases and 41 malignant cases were correctly diagnosed by CEUS in this study, and the sensitivity, specificity, accuracy, positive predictive value and negative predictive value of CEUS for diagnosing malignant thyroid lesions were 83.7%, 64.5%, 76.3%, 78.9% and 71.4%, respectively. The significant ring of hyper-enhancement of benign nodules especially thyroid adenomas, is because most benign nodules have a capsule with blood vessels distributed along the sub-capsule. For other types of benign nodules, the enhancement of the halo was almost synchronized with the nodule, which is attributed to the fact that benign nodules have a thin halo with blood vessels evenly distributed within and without compression. In addition, the “partial volume effect” also affects the enhancement of the thin halo, causing synchronous enhancement with the surrounding thyroid tissue, which provides a basis for the diagnosis of benign thyroid nodules [25]. In contrast to benign nodules, the flow in the halo of malignant nodules is mainly dotted distributed, which contributed to the fact that 98.1% of malignant nodules were hypo-enhanced on CEUS in this study. This is mainly due to changes such as edema, mucinous degeneration, inflammation, and fibrosis around the nodules, resulting in a decrease and compression of the vascular component in the halo.

Ultrasound-guided FNA cytology has become the most direct method for diagnosing the nature of thyroid nodules because it is safe, accurate, rapid, and cost-effective, but it is still influenced by the operator and the satisfaction with the material obtained, and there were even discordant FNAC reports were distributed, which in turn required a repeat of the FNAC [26–28]. TH Yin [29] et al. reported that high-risk factors such as cystic nodules, nodules with hemorrhage and suspicious degenerating nodules may reduce specimen acquisition during FNA because viable tissue is rarely present in cystic nodules or it is difficult to distinguish between viable and inactive tissue with conventional ultrasound. In the present study, FNA diagnosed 20 benign and 50 malignant cases correctly, and the sensitivity, specificity, accuracy, positive predictive value and negative predictive value of FNA in diagnosing malignant thyroid lesions were 86.2%, 90.9%, 82.5%, 96.2% and 71.4%, respectively, higher than the results of CEUS, but still lower than the results of CEUS combined with FNA, which were 94.2%, 89.3%,92.5%, 94.2% and 89.3%, respectively. This is because the operator is unable to determine the distribution of the active component of the nodule during the process of FNA; although multidirectional and multiangle punctures can improve the effectiveness of extraction, there is still some uncertainty. However, with the assistance of CEUS, the operator can effectively avoid necrotic areas without vascular distribution and perform focused puncture on suspicious areas, to improve the efficiency and accuracy of the FNA results. The AUC of CEUS, FNA and CEUS combined with FNA were 75.1%, 86.3%, and 91.8%, respectively, further confirming that the combined method can effectively improve the diagnostic efficacy of nodules, which is consistent with the findings of TH Yin [29].

Although most thyroid cancers especially papillary carcinoma generally have slow disease progression with low mortality rates and a favorable long-term prognosis, the presence of cervical lymph node metastasis remains a significant risk factor for both local recurrence and distant metastasis [30–33]. Metastasis refers to the continued growth of tumor cells from the primary site to other sites through lymphatic vessels and blood vessels [34]. However, if the tumor is restrained or compressed by external force during growth, the expansive growth of the tumor will be inhibited, and the blood vessels or lymphatic vessels around the tumor will be blocked due to compression, which can theoretically inhibit the metastasis of tumor cells to a certain extent. In this study we summarized the pathology of the halo of benign and malignant nodules, and found that the main component of the halo of malignant nodules is fibrous connective tissue, which is caused by malignant tumors oppressing the surrounding tissues during the growth process and repeated inflammation. As the fibrous component of the acoustic corona increases, the vascular and lymphatic components further decrease, which reduces the possibility of tumor cell metastasis through lymphatic vessels. In addition, the increase in fiber content will also cause compression and squeezing on the lymphatic vessels, blocking the metastasis of tumor cells through the lymphatic vessels. Zheng et al. revealed that the pathological annulus fibrosus in the nonhyperechoic halo group often appeared incomplete, uneven, thin, or absent, and had a high rate of metastasis [35]. In fact, in the present study, only 13 out of 110 (11.5%) patients with malignant tumors had cervical lymph node metastases, and the findings are consistent with the literature. Analysis of the causes revealed that the halo of all 13 nodules was uneven in thickness, with the thinnest being only 3.4 mm, and their pathology suggested weak fibrous tissue, which may be an important cause of metastasis. Therefore, it is reasonable to speculate that malignant thyroid nodes with halos are less likely to have cervical lymph node metastasis than malignant nodes without halos. We will also increase the number of cases in the follow-up study to further test this speculation.

In summary, in the present study we found that malignant thyroid nodules had thicker and heterogeneous acoustic halos with poor integrity. The halo of malignant nodules was predominantly without or with little blood flow and showed hypo-enhancement on CEUS. The diagnostic efficacy of CEUS combined with FNA was significantly higher than that of CEUS. The pathological features of the halo were infiltrative inflammatory cells and a large amount of reactive hyperplastic fibrous tissue. In addition, we predicted a reduced risk of lymph node metastasis in malignant nodes with halos.

The present study has some limitations. Due to the limited number of cases, a larger sample is needed to verify the reliability of the findings of this study. In addition, because ultrasound is an examination that is greatly influenced by a variety of objective factors and subjective judgments, the measurement of the acoustic halo is influenced by many factors, such as instrument clarity, operating technique, scanning cross-section, and clinical experience. Therefore, it is necessary to improve the reproducibility and double-blindness of the study to obtain more reliable results.

## Electronic supplementary material

Below is the link to the electronic supplementary material.

Supplementary Material 1

## Data Availability

No datasets were generated or analysed during the current study.
