# Supplementary material for: Sonographic characteristics of thyroid nodules with a Halo
Source: Thyroid Res. 2024 Oct 1;17:20. doi: 10.1186/s13044-024-00208-5 (PMC11443876; doi:10.1186/s13044-024-00208-5)
Supplement: Supplementary file 1 — Supplementary Material 1 [file 13044_2024_208_MOESM1_ESM.docx]

**Supplementary table 1** Baseline characteristics of group I(CEUS) and III(CEUS+FNA)

| **Variables** | Total (n=80) | Malignant | Benign | *P* |
| --- | --- | --- | --- | --- |
| **age, Mean ± SD** | 42.0 ± 11.4 | 43.7 ± 12.0 | 38.7 ± 9.5 | 0.058 |
| **Gender, n (%)** |  |  |  | 0.03 |
| Male | 44 (55.0) | 24 (46.2) | 20 (71.4) |  |
| Female | 36 (45.0) | 28 (53.8) | 8 (28.6) |  |
| **Characteristics of nodules** |  |  |  |  |
| **Echogenicity, n (%)** |  |  |  | 0.979 |
| Hypo-echoic | 23 (28.7) | 19 (36.5) | 6 (21.4) |  |
| Iso or hyper-echoic | 57 (71.2) | 33 (63.5) | 22(78.6) |  |
| **Margin, n (%)** |  |  |  | < 0.001 |
| Well-defined | 35 (43.8) | 12 (23.1) | 23(82.1) |  |
| Ill-defined | 45 (56.2) | 40 (76.9) | 5 (17.9) |  |
| **Calcification, n (%)** |  |  |  | < 0.001 |
| Absent or macro-calcification | 27 (33.8) | 2 (3.8) | 25 (89.3) |  |
| Micro-calcification | 53 (66.2) | 50 (96.2) | 3 (10.7) |  |
| **Shape, n (%)** |  |  |  | < 0.001 |
| Taller than wide | 37 (46.2) | 34 (65.4) | 3 (10.7) |  |
| Wider than tall | 43 (53.8) | 18 (34.6) | 25 (89.3) |  |
| **Flow, n (%)** |  |  |  | < 0.001 |
| Dotted | 41 (51.2) | 37 (71.2) | 4 (14.2) |  |
| Striped | 27 (33.8) | 15 (28.8) | 12 (42.9) |  |
| Circular | 10 (15.0) | 0 (0) | 12 (42.9) |  |
| **Enhancement mode, n (%)** |  |  |  | < 0.001 |
| Hyper or iso-enhancement | 46 (57.5) | 21 (40.4) | 25 (89.3) |  |
| Hypo-enhancement | 34 (42.5) | 31 (59.6) | 3 (10.7) |  |
| **Uniformity , n (%)** |  |  |  | 0.003 |
| Heterogeneous | 49 (61.3) | 38 (73.1) | 11 (39.3) |  |
| Homogeneous | 31 (38.8) | 14 (26.9) | 17 (60.7) |  |
| **Regularity, n (%)** |  |  |  | < 0.001 |
| Even | 33 (41.2) | 8 (15.4) | 25 (89.3) |  |
| Uneven | 47 (58.8) | 44 (84.6) | 3 (10.7) |  |
| **Integrity, n (%)** |  |  |  | < 0.001 |
| Yes | 34 (42.5) | 9 (17.3) | 25 (89.3) |  |
| No | 46 (57.5) | 43 (82.7) | 3 (10.7) |  |
| **Characteristics of halo** |  |  |  |  |
| **Thickness (mm), Mean± SD** | 1.11 ± 0.54 | 1.35 ± 0.50 | 0.66 ± 0.28 | < 0.001 |
| **Thickness>1mm, n (%)** |  |  |  | < 0.001 |
| Yes | 61 (76.2) | 50 (96.2) | 11 (39.3) |  |
| No | 19 (23.8) | 2 (3.8) | 17 (60.7) |  |
| **Flow , n (%)** |  |  |  | < 0.001 |
| Dotted/Striped | 68 (85.0) | 50 (96.2) | 18 (64.3) |  |
| Ring | 12 (15.0) | 2 (3.8) | 10 (35.7) |  |
| **Enhancement mode, n (%)** |  |  |  | < 0.001 |
| Hyper or iso-enhancement | 27 (33.8) | 1 (1.9) | 26 (92.9) |  |
| Hypo-enhancement | 53 (66.2) | 51 (98.1) | 2 (7.1) |  |
| **TI-RADS, n (%)** |  |  |  | < 0.001 |
| 3 | 19 (23.8) | 0 (0) | 19 (67.9) |  |
| 4a | 17 (21.2) | 10 (19.2) | 7 (25) |  |
| 4b | 19 (23.8) | 17 (32.7) | 2 (7.1) |  |
| 4c | 18 (22.5) | 18 (34.6) | 0 (0) |  |
| 5 | 7 ( 8.8) | 7 (13.5) | 0 (0) |  |
| **Bethesda, n (%)** |  |  |  | < 0.001 |
| II | 18 (22.5) | 0 (0) | 18 (64.3) |  |
| III | 11 (13.8) | 4 (7.7) | 7 (25) |  |
| IV | 29 (36.2) | 26 (50) | 3 (10.7) |  |
| V | 22 (27.5) | 22 (42.3) | 0 (0) |  |

**Supplementary table 2** Baseline characteristics of group II (FNA)

| **Variables** | Total (n=80) | Malignant | Benign | *P* |
| --- | --- | --- | --- | --- |
| **age, Mean ± SD** | 42.51 ± 9.27 | 43.60 ± 8.92 | 39.64 ± 9.77 | 0.088 |
| **Gender, n (%)** |  |  |  | 1 |
| Male | 10(12.5) | 7(12.1) | 3(13.6) |  |
| Female | 70 (87.5) | 51 (87.9) | 19 (86.4) |  |
| **Characteristics of nodules** |  |  |  |  |
| Echogenicity, n (%) |  |  |  | 1 |
| Hypo-echoic | 8(10) | 46(79.3) | 2(9.1) |  |
| Iso or hyper-echoic | 72 (90) | 12(21.7) | 20 (90.9) |  |
| Margin, n (%) |  |  |  | < 0.001 |
| Well-defined | 18 (22.5) | 3 (5.2) | 15 (68.2) |  |
| Ill-defined | 62 (77.5) | 55 (94.8) | 7 (31.8) |  |
| Calcification, n (%) |  |  |  | 1 |
| Absent or macro-calcification | 66 (82.5) | 48 (82.8) | 18 (81.8) |  |
| Micro-calcification | 14 (17.5) | 10 (17.2) | 4 (18.2) |  |
| Shape, n (%) |  |  |  | < 0.001 |
| Taller than wide | 58 (72.5) | 54 (93.1) | 4 (18.2) |  |
| Wider than tall | 22 (27.5) | 4 (6.9) | 18 (81.8) |  |
| Blood of the nodules, n (%) |  |  |  | < 0.001 |
| Dotted | 58 (72.5) | 55 (94.8) | 3 (13.6) |  |
| Striped | 22 (27.5) | 3 (5.2) | 17 (78.3) |  |
| Circular | 2(2.5) | 0(0) | 2(9.1) |  |
| **Characteristics of halo** |  |  |  |  |
| **Thickness (mm), Mean± SD** | 1.38 ± 0.55 | 1.58 ± 0.49 | 0.87 ± 0.31 | < 0.001 |
| Thickness>1mm, n (%) |  |  |  | < 0.001 |
| Yes | 44 (55.0) | 41 (70.7) | 3 (13.6) |  |
| No | 36 (45.0) | 17 (29.3) | 19 (86.4) |  |
| Flow, n (%) |  |  |  | 0.7 |
| Dotted/Striped | 71 (88.8) | 52 (89.7) | 19 (86.4) |  |
| Ring | 9 (11.2) | 6 (10.3) | 3 (13.6) |  |
| **Bethesda, n (%)** |  |  |  | < 0.001 |
| II | 16 (20.0) | 1 (1.7) | 15 (68.2) |  |
| III | 9 (11.2) | 5 (8.6) | 4 (18.2) |  |
| IV | 34 (42.5) | 31 (53.4) | 3 (13.6) |  |
| V | 21 (26.2) | 21 (36.2) | 0 (0) |  |
